# Severe ineffective erythropoiesis discriminates prognosis in myelodysplastic syndromes: analysis based on 776 patients from a single centre

**DOI:** 10.1038/s41408-020-00349-4

**Published:** 2020-08-14

**Authors:** Huijun Huang, Changlu Xu, Jie Gao, Bing Li, Tiejun Qin, Zefeng Xu, Sirui Ren, Yudi Zhang, Meng Jiao, Shiqiang Qu, Lijuan Pan, Naibo Hu, Jinqin Liu, Wenyu Cai, Yingnan Zhang, Dan Wu, Peihong Zhang, Robert Peter Gale, Gang Huang, Jiaxi Zhou, Lihong Shi, Zhijian Xiao

**Affiliations:** 1 MDS and MPN Centre, Institute of Hematology and Blood Diseases Hospital, Chinese Academy of Medical Sciences & Peking Union Medical College, Tianjin, China; 2 State Key Laboratory of Experimental Hematology, Institute of Hematology and Blood Diseases Hospital, Chinese Academy of Medical Sciences & Peking Union Medical College, Tianjin, China; 3 National Clinical Research Centre for Blood Diseases, Institute of Hematology and Blood Diseases Hospital, Chinese Academy of Medical Sciences & Peking Union Medical College, Tianjin, China; 4 Pathology Centre, Institute of Hematology and Blood Diseases Hospital, Chinese Academy of Medical Sciences & Peking Union Medical College, Tianjin, China; 5 Haematology Section, Division of Experimental Medicine, Department of Medicine, Imperial College London, London, UK; 6 Divisions of Pathology and Experimental Hematology and Cancer Biology, Cincinnati Children’s Hospital Medical Center, Cincinnati, OH USA

**Keywords:** Myelodysplastic syndrome, Prognosis

## Abstract

The underlying mechanisms and clinical significance of ineffective erythropoiesis in myelodysplastic syndromes (MDS) remain to be fully defined. We conducted the ex vivo erythroid differentiation of megakaryocytic-erythroid progenitors (MEPs) from MDS patients and discovered that patient-derived erythroblasts exhibit precocity and premature aging phenotypes, partially by inducing the pro-aging genes, like *ERCC1*. Absolute reticulocyte count (ARC) was chosen as a biomarker to evaluate the severity of ineffective erythropoiesis in 776 MDS patients. We found that patients with severe ineffective erythropoiesis displaying lower ARC (<20 × 10^9^/L), were more likely to harbor complex karyotypes and high-risk somatic mutations (*p* < 0.05). Lower ARCs are associated with shorter overall survival (OS) in univariate analysis (*p* < 0.001) and remain significant in multivariable analysis. Regardless of patients of lower-risk who received immunosuppressive therapy or higher-risk who received decitabine treatment, patients with lower ARC had shorter OS (*p* < 0.001). Whereas no difference in OS was found between patients receiving allo-hematopoietic stem cell transplantations (Allo-HSCT) (*p* = 0.525). Our study revealed that ineffective erythropoiesis in MDS may be partially caused by premature aging and apoptosis during erythroid differentiation. MDS patients with severe ineffective erythropoiesis have significant shorter OS treated with immunosuppressive or hypo-methylating agents, but may benefit from Allo-HSCT.

## Introduction

Myelodysplastic syndromes (MDS), a heterogeneous clonal myeloid neoplasm characterized by ineffective hematopoiesis leading to cytopenia(s) and a risk of leukemia transformation^1^, is one of the most frequently encountered acquired bone marrow failure (BMF) syndromes in adults. Genetic and epigenetic changes that affect hematopoietic stem cells (HSCs) and alterations in the hematopoietic niche resulting in degeneration and apoptosis in hematopoietic stem and progenitor cells (HSPCs) mainly contribute to ineffective hematopoiesis^2,3^.

Ineffective erythropoiesis refers to the inability to produce adequate red blood cells (RBCs) with increased dysplastic erythroid precursors in the BM. Anemia is the most common symptom in MDS which correlates with ineffective erythropoiesis. Previous studies suggested that the deficiencies of erythroid lineage of MDS patients were more pronounced than other myeloid lineages^4^, highlighting the widespread ineffective erythropoiesis in MDS. Ineffective erythropoiesis in MDS has long been attributed to the differentiation arrest and increased apoptosis of erythroid precursors induced by different mechanisms, such as genetic lesions originating in HSCs, excessive proinflammatory cytokines and immune disorders^3,5–7^. Recent investigations also implicated activation of the NLRP3 inflammasome in HSPCs as a critical convergent signal in MDS with consequential pyroptotic cell death^8,9^. However, other potential mechanisms responsible for defective erythropoiesis are still elusive. Moreover, a reliable biomarker to accurately reflect the severity of BM ineffective erythropoiesis has been lacking so far and its prognostic significance remains to be carefully defined.

In this study, the HSCs and megakaryocytic-erythroid progenitors (MEPs) in the BM mononuclear cells (BMMNCs) of MDS patients (*n* = 31) were measured by flow cytometry and in vitro colony-forming unit cell (CFU-C) were assayed in 612 MDS patients, and we found that the percentage of HSCs and MEPs in the BMMNCs, the numbers of burst-forming unit-erythroid (BFU-E) and colony forming unit-erythroid (CFU-E) of MDS patients were sifgnificantly reduced compared with health controls. The ex vivo erythroid differentiation of MEPs derived from MDS patients (*n* = 31) were conducted and we found the occurrence of precocity in erythroid progenitor and precursor cells of MDS patients which were prone to undergo subsequent premature aging and apoptosis. As the absolute reticulocyte count (ARC) can reflect the erythropoietic activity of BM properly^10^, we therefore chose ARC as a biomarker to evaluate the severity of ineffective erythropoiesis. Here, we adopted a cut-off of ARC less than 20 × 10^9^/L, which is one of the diagnostic criteria of severe aplastic anemia (SAA) in guidelines recommended by the British Committee for Standards in Haematology (BCSH)^11^, to discriminate the extent of ineffective erythropoiesis. Based on this, a cohort of 776 newly diagnosed MDS patients in our center were divided into two groups and their clinical outcomes were investigated. Our data showed that a severe reduced ARC (<20 × 10^9^/L) acted as a powerful independent prognostic factor in MDS which could not be reversed by immunosuppressive treatments in Revised International Prognostic Scoring System (IPSS-R) lower-risk patients and hypo-methylating agents in IPSS-R higher-risk patients, but had no impact on the survival of patients in both IPSS-R categories when received allo-hematopoietic stem cell transplantation (Allo-HSCT).

## Methods

### Patients and samples

BM samples and umbilical cord blood were harvested after informed consent approved by the Ethical Committee on Medical Research at Institute of Hematology and blood disease hospital. In detail, BM samples were collected from 31 newly diagnosed MDS patients according to the 2016 revised criteria of the World Health Organization (WHO)^12^, and 12 healthy donors for the cellular studies and transcriptome sequencing analysis. Clinical features of these patients are shown in Table S1. Additionally, a total of 776 consecutive newly diagnosed MDS patients at our center, from December 2011 to December 2018, constituted the clinical cohort. BM and Blood samples were obtained from all patients at diagnosis. Wright-Giemsa-stained BM and Blood smears were reviewed according to the 2016 revised criteria by two expert pathologists (W Cai, P Zhang). Detailed characteristics of all patients are shown in Table 1. A total of 678 (87.4%) patients had evaluable karyotypes. 612 (78.9%) patients experienced CFU-C assays, the reference of normal average (Mean±SD) at our lab were: BFU-E 31 ± 6, CFU-E 81 ± 12 and colony forming unit-granulocyte/macrophage (CFU-G/M) 22 ± 7^13^. 553 patients were tested for serum erythropoietin (EPO) levels at diagnosis. DNA derived from BMMNCs from all patients underwent the targeted 112-gene sequencing at diagnosis^14^. The prognostic categories were evaluated with the IPSS-R^15^. An IPSS-R scores ≤ 3.5 were classified into a lower-risk cohort while >3.5 into a higher-risk cohort^16^. Data of treatments were available for 575 patients. Among them, 48 (8.3%) received erythropoietin with or without G-CSF, RBC and/or platelet transfusions; 303 (52.7%) patients received immunosuppressive drugs using cyclosporine combined with danazol (±thalidomide); 91 (15.8%) received decitabine; 38 (6.6%) received chemotherapy using aclacinomycin or homoharringtonine combined with cytarabine and granulocyte-colony stimulating factor (G-CSF; termed CAG or HAG regimen), idarubicin or daunorubicin combined with cytarabine (IA or DA) or melphalan; 74 (12.9%) received Allo-HSCT, and 21 (3.7%) received traditional Chinese medicines. Follow-up data were available for 687 (88.5%) patients with a median follow-up of 17 months (range, 2–87 months). Overall survival (OS) was calculated from the date of diagnosis to the date of last follow-up or death. All subjects provided informed consent in compliance with the Declaration of Helsinki.

**Table 1:** Clinical and laboratory characteristics of 776 patients with MDS.

Characteristics	ARC < 20 × 10^9^/L (*N* = 169)	ARC ≥ 20 × 10^9^/L (*N* = 607)	Total (*N* = 776)	*p* value
Sex, *n* (%)				0.046
Male	119 (70.4%)	375 (61.8%)	494 (63.7%)	
Female	50 (29.6%)	232 (38.2%)	282 (36.35%)	
Age, median (range), y	56 (16–83)	52 (14–83)	54 (14–83)	0.032
Age ≥60 years, *n* (%)	73 (43.2%)	199 (32.8%)	272 (35.1%)	0.014
WHO classification 2016*, n* (%)				0.101
MDS-SLD	4 (2.4%)	30 (4.9%)	34 (4.4%)	
MDS-RS-SLD	4 (2.4%)	18 (3.0%)	22 (2.8%)	
MDS-MLD	71 (42.0%)	307 (50.6%)	378 (48.7%)	
MDS-RS-MLD	5 (3.0%)	10 (1.6%)	15 (1.9%)	
MDS-EB1	39 (23.1%)	114 (18.8%)	153 (19.7%)	
MDS-EB2	41 (24.3%)	104 (17.1%)	145 (18.7%)	
MDS with isolated del (5q)	0	7 (1.2%)	7 (0.9%)	
MDS-U	5 (3.0%)	17 (2.8%)	22 (2.8%)	
Hb, median (range), g/L	64 (31–138)	83 (38–155)	78 (31–155)	<0.001
WBC, median (range), ×10^9^/L	2.51 (0.71–21.17)	2.83 (0.61–20.42)	2.75 (0.61–21.17)	0.007
ANC, median (range), ×10^9^/L	0.99 (0.04–13.18)	1.19 (0–17.37)	1.15 (0–17.37)	0.002
PLT, median (range), ×10^9^/L	51 (2–536)	63 (2–694)	60 (2–694)	0.012
BM erythroblasts, median (range), %	20 (0–75)	35 (0–92.5)	31 (0–92.5)	<0.001
Sum of proerythroblast and basophilic erythroblast(E1), median (range), %	1 (0–30)	1.5 (0–18.5)	1.5 (0–30)	0.009
Sum of polychromatic and orthochromatic erythroblast (E2), median (range), %	17.5 (0–67)	32 (0–88)	29 (0–88)	<0.001
Ratio of E1 to E2, median (range)	0.052 (0–1.75)	0.05 (0–2)	0.05 (0–2)	0.305
BM blast, median (range), %	3.5 (0–19.5)	2.5 (0–19.5)	2.5 (0–19.5)	0.005
IPSS-R karyotype, *n* (%), *N* = 678				<0.001
Very good	1 (0.7%)	7 (1.3%)	8 (1.2%)	
Good	69 (48.6%)	313 (58.4%)	382 (56.3%)	
Intermediate	26 (18.3%)	138 (25.7%)	164 (24.2%)	
Poor	11 (7.7%)	29 (5.4%)	40 (5.9%)	
Very poor	35 (24.6%)	49 (9.1%)	84 (12.4%)	
Complex karyotype, *n* (%)	39 (27.5%)	68 (12.7%)	107 (15.8%)	<0.001
IPSS-R risk group, *n* (%)*, N* = 678				<0.001
Very low	0	21 (3.9%)	21 (3.1%)	
Low	21 (14.8%)	148 (27.6%)	169 (24.9%)	
Intermediate	41 (28.9%)	175 (32.6%)	216 (31.9%)	
High	30 (21.1%)	122 (22.8%)	152 (22.4%)	
Very high	50 (35.2%)	70 (13.1%)	120 (17.7%)	
IPSS-R two groups, *n* (%)				<0.001
Lower-risk	40 (28.2%)	245 (45.7%)	285 (42.0%)	
Higher-risk	102 (71.8%)	291 (54.3%)	393 (58.0%)	

*MDS* myelodysplastic syndrome, *ARC* absolute reticulocyte count, *MDS-SLD* MDS with single lineage dysplasia, *MDS-RS-SLD* MDS with ring sideroblasts with single lineage dysplasia, *MDS-MLD* MDS with multilineage dysplasia, *MDS-RS-MLD* MDS with ring sideroblasts with multilineage dysplasia, *MDS-EB1* MDS with excess blasts-1, *MDS-EB2* MDS with excess blasts-2, *MDS-U* MDS unclassifiable, *Hb* haemoglobin, *WBC* white blood coun, *ANC* absolute neutrophil count, *PLT* platelet count, *BM* bone marrow, *IPSS-R* Revised International Prognostic Scoring System.

### Fluorescence-activated cell sorting analysis

The procedure for Fluorescence-activated cell sorting (FACS) staining was performed as described previously^17^. For detailed information of sorting strategy of HSCs and MEPs, please refer to supplementary methods.

### Ex vivo erythroid differentiation

Purification of CD34^+^ cells from BM of MDS patients and healthy donors or from human umbilical cord blood was carried out as previously^18^. The purified CD34^+^ cells or FACS sorted MEPs were induced ex vivo differentiation toward erythroid lineage as previously described^18^. Please refer to Supplementary Methods for details.

### 293T and K562 cell culture

293T and K562 cells were obtained from the American Type Culture Collection (ATCC) and cultured according to ATCC recommendations. Erythroid differentiation of K562 cells was induced with hemin (30 μM) for 3 days.

### Wright-Giemsa and benzidine staining

Cell morphology was analyzed by Wright-Giemsa staining (Baso, Cat# BA-4017). Hemoglobin synthesis was assessed by benzidine staining^19^. The benzidine-positive cells were stained in brown.

### Measurement of cellular aging

Senescent cell detecting was performed using a β-galactosidase staining Kit (Beyotime biotechnology), according to the manufacturer’s instructions. Senescent cells will be stained blue.

### RNA-Seq and Gene sets enrichment analysis

Library construction and data processing were performed by Novogene (Beijing, China) as previously described^18^. The gene expression matrix was used to conduct Gene sets enrichment analysis (GSEA). RNA sequencing data has been uploaded to the Gene Expression Omnibus database. If there is any reasonable need, please contact the corresponding author for the accession number. Detailed information were described in supplementary methods.

### RNA extraction and qRT-PCR

Total RNA extraction and quantitative real-time PCR (qRT-PCR) were performed as mentioned before^18^. Primer sequences used for qRT-PCR are shown in Table S2.

### Plasmid construction, lentivirus production, and infection

The short hairpin RNA (shRNA) sequences targeting *ERCC1* were cloned into lentiviral shRNA expression vector pSIH-H1, containing a GFP expression marker. Lentiviruses were packaged using ViraPower Lentiviral Packaging system (Invitrogen). For infection, K562 cells and primary erythroid cells at day 4 of differentiation were incubated with lentiviruses for 12 h before washing away the excess virus. The shRNA sequences are listed in Table S2.

### Western blotting

Detailed information for western blotting (WB) were described before^18^. Specially, antibody to *ERCC1* (Proteintech) was diluted to a final concentration of 1:300 and α-Tubulin (Abcam) was diluted to 1:5000, which served as the loading control.

### Statistical analysis

Categorical variables were analyzed by the Fisher test and continuous variables by Mann–Whitney U test or Kruskal–Wallis analysis, as appropriate. Survival analysis was carried out with the Kaplan–Meier method and compared with the log-rank test. Cox proportional hazard regression model was used for multivariable analysis. Two-tailed *p* values < 0.05 were considered significant. All the analyses were conducted using SPSS, version 25.0.

## Results

### Erythropoietic activity was impaired in BM of MDS patients

The HSCs and MEPs in the BMMNCs of MDS patients (*n* = 31) and healthy donors (n = 12) were measured by flow cytometry with the markers of Lin^-^CD34^+^CD38^-^CD45RA^-^CD123^-^ for HSC and Lin^-^CD34^+^CD38^+^CD45RA^-^CD123^-^ for MEP (Fig. S1)^17^. Both percentage of HSCs and MEPs in Lin^−^ cells were lower in MDS patients compared with healthy controls (*p* = 0.001; *p* = 0.084; Fig. S2A, B). As a consequence of the shortage of HSPCs, we observed the low production of erythroid progenitors, including BFU-E and CFU-E (Fig. S2C). Next, to investigate the capacity of terminal erythroid differentiation of MDS patients, we analyzed the percentage of pro-, basophilic, polychromatic and orthochromatic erythroblasts in BM of 776 MDS patients, respectively. The median BM erythroblast percentage was 31% (0–92.5%, Table 1), of which the frequency of relative immature proerythroblast and basophilic erythroblast (E1) comprised 1.5% (0–30%, Table 1), and the more differentiated polychromatic and orthochromatic erythroblast (E2) constituted 29% (0–88%, Table 1). The median ratio of E1 to E2 was 0.05 (0–2, Table 1), which was higher than that in chronic aplastic anemia patients reported in our previous study^20^, revealing the blockage of erythroid differentiation to a certain extent in MDS. Collectively, BM erythropoietic activity was impaired in MDS patients due to both quantitative and qualitative defects of erythroid precursors.

### Precocity and premature aging occurring in patient-derived erythroid cells may be involved in ineffective erythropoiesis

**Fig. 1:**
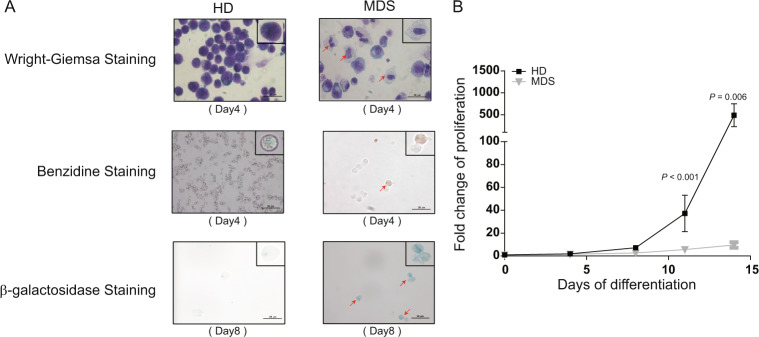
Inducing ex vivo erythroid lineage differentiation from MDS patients derived megakaryocytic-erythroid progenitors (MEPs). **a** Representative cytospin staining images of erythroid cells derived from MDS patients and healthy donors (HD) cultured for 4 days and 8 days. The cells indicated by red arrows refer to dysplastic erythrocytes, benzidine-positive and β-galactosidase-positive cells, respectively, in MDS patients which suggested the precocity and prematuring aging in erythroid differentiation of MDS. Scale bar = 20 μm. **b** Cell proliferation curve was drawn according to cell counts at each stage.

To elucidate the underlying mechanism of the ineffective erythropoiesis, MEPs from MDS patients (*n* = 31) and healthy donors (*n* = 12) were purified and induced differentiation towards erythroid lineage ex vivo for 18 days. As shown in Fig. 1a, during the process of differentiation, compared to the regular cell morphology, we observed the aberrant differentiated cells with vacuolate cytoplasm and deformed cell membranes in MDS patients on day 4 of differentiation. The synthesis of hemoglobin is vital for functional maturity of erythroid cells. Here, we conducted the Benzidine staining to monitor globin synthesis in patient-derived erythroid cells at various differentiation stages. Intriguingly, we detected the benzidine-positive cells (brown, indicated by arrow, 4.5%) as early as day 4 of differentiation in cells from patients, when the majority of cells in normal controls are lineage progenitors and incapable of producing hemoglobin (Fig. 1a). Such a trend of more active hemoglobin synthesis in MDS-derived cells persisted throughout the differentiation (44 vs. 26% at day 11 and 54 *vs*. 40% at day 14) except the final stage (day 18) (Fig. S3A, B), despite they did not reach the statistical significance (*p* > 0.05) probably due to the limited sample size. These results suggested that precocity, especially from a functional perspective, may occur during erythroid differentiation in MDS patients. We next utilized senescence-associated β-galactosidase staining, by which the aging cells could be stained blue. We observed that on average 43.2% of patient-derived cells from day 8 of culture were dyed blue, which was much higher than that of healthy donors (43.2 vs. 10%, *p* = 0.007; Fig. 1a and Fig. S3C). This result confirmed a premature aging of erythroid precursors in MDS-derived cells. Inherited with such intrinsic disorders, the cell proliferation curve, which was drawn according to the numbers of cells at each stage, showed that the proliferation ability of erythroid cells derived from MEPs of MDS patients was significantly decreased when compared with normal controls (Fig. 1b).

### The aberrant molecular pathways account for the premature aging of erythroid cells from MDS patients

To further explore the molecular mechanisms of the ineffective erythropoiesis in MDS, we next conducted low-input RNA-seq analysis in highly purified HSCs (20–200 cells), MEPs (20–200 cells) and cultured erythroid cells at day 4 and 8 of differentiation from purified MEPs. GSEA analysis showed that several signaling pathways were significantly different between MDS patients and healthy donors (Fig. S4).

**Fig. 2:**
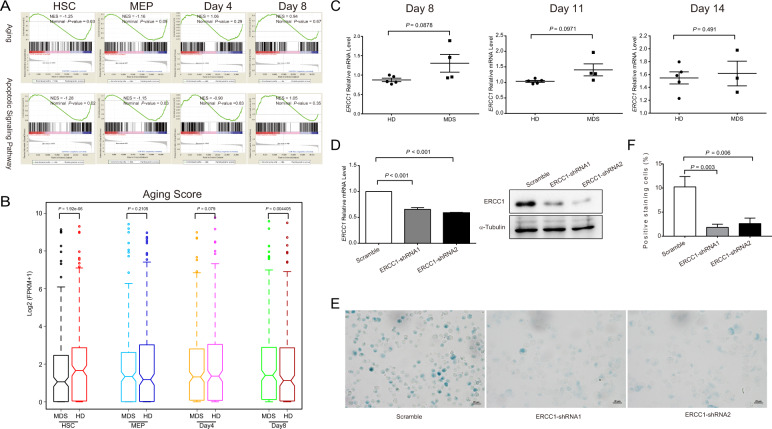
Aging-associated genes were upregulated in MDS-derived erythroid cells during ex vivo differentiation. **a** Gene set enrichment analysis (GSEA) of aging and apoptotic_signaling_pathway in hematopoietic cells at various differentiation stages from MDS patients and HD, respectively. Normalized Enrichment Score (NES) and Nominal *p*-value are shown in each plot. **b** Aging Score was calculated based on FPKM of aging genes. Wilcoxon Signed-rank Test (Paired) was used to analyze the statistical significance and *p* values are shown in the plot. **c** The expression of *ERCC1*, one of the aging-associated genes, at day 8, 11 and 14 of erythroid differentiation in MDS patients and HD by qRT-PCR. **d** The expression of *ERCC1* was measured by qRT-PCR and WB, respectively, in primary erythroid cells at day 8 of differentiation after lentiviral-mediated knockdown. **e** The representative β-galactosidase staining images of day 8-differentiated primary erythroid cells in *ERCC1* diminished or scramble control cells (Scale bar = 20 μm). **f** The bar graph showing the percentage of β-galactosidase positive cells in control and *ERCC1*-diminished cells at day 8 of erythroid differentiation derived from cord blood CD34^+^ cells.

To further provide the molecular evidence of premature aging in MDS patients, we particularly examined the dynamic alteration of gene expression associated with aging. As shown in Fig. 2a, the aging pathway was gradually upregulated in MDS patients from HSCs to erythroid cells at day 8, which was in line with the quantitative analysis (Fig. 2b). The aberrant expression of aging-associated genes in patients was identified as well (Fig. S5). For instance, we detected *ERCC1*, which is proved to be related to glomerular aging and Parkinson’s disease^21,22^; *EIF2S1*, which is the target gene of Nuclear respiratory factor 1 (*NRF1*). *NRF1* is a transcription factor that activates the expression of a wide range of genes associated with neurodegenerative diseases^23^; and *SRR*, which is one of single-nucleotide polymorphisms (SNPs), may contribute to age-related cognitive decline^24^. Among them, the trend of higher expression of *ERCC1* was further confirmed in MDS-derived erythroid cells during ex vivo differentiation by qRT-PCR (Fig. 2c). Additionally, consistent with previous studies, we also detected the active apoptotic signaling pathway in MDS patients (Fig. 2a)^25,26^.

To unravel the role of *ERCC1* during hematopoietic aging, we infected lentiviral-mediated *ERCC1*-targeting shRNAs into K562 cells or primary erythroid cells differentiated for 4 days from cord blood CD34^+^ cells. The reduced expression of *ERCC1* was confirmed on mRNA level by qRT-PCR and protein level by WB in both primary erythroid cells and K562 cells, respectively (Figs. 2d and S6A). In *ERCC1* diminished cells, we found that there were much less β-galactosidase positive cells in primary erythroid cells at day 8 of differentiation (Fig. 2e, f), which was in line with hemin-induced K562 cells (Fig. S6B, C). Collectively, these results imply *ERCC1* promotes the aging process of erythroid cells. Additionally, as the result of its pro-aging effects, we also found *ERCC1* deficient K562 cells showed stronger proliferation ability compared with scramble control during hemin-induced erythroid differentiation (Fig. S6D). Therefore, the induced *ERCC1* expression might reflect the premature aging of the MDS-derived erythroid precursors.

### Severe reduced ARC is strongly correlated with high-risk cytogenetic and molecular genetic aberrations

The main feature of MDS is stem-cell-derived clonal myelopoiesis with altered proliferation and differentiation leading to dyshematopoiesis. Genetic defects play a fundamental role in pathogenesis of MDS^27^. To determine the association between genetic aberrations and dyserythropoiesis, we analyzed results of chromosome abnormalities and somatic gene mutations in 776 MDS patients stratified by levels of ARC given that ARC serves as an indicator reflecting BM erythropoietic activity. Overall, the median ARC level of all patients was 43.1 (0.8–660) × 10^9^/L. Distributions of ARC among different WHO diagnostic categories and IPSS-R risk groups are shown in Fig. S7. The 776 patients were divided into two cohorts: lower ARC (<20 × 10^9^/L, *n* = 169) and higher ARC (≥20 × 10^9^/L, *n* = 607) cohorts. Patients in the lower ARC cohort had lower hemoglobin levels (64 g/L vs. 83 g/L; *p* < 0.001; Table 1), lower white blood cell (WBC) count (2.51 × 10^9^/L vs. 2.83 × 10^9^/L; *p* = 0.007; Table 1), lower neutrophil absolute count (0.99 × 10^9^/L *vs*. 1.19 × 10^9^/L; *p* = 0.002; Table 1), lower platelet count (51 × 10^9^/L vs. 63 × 10^9^/L; *p* = 0.012; Table 1), lower BM erythroblast percentage (20% vs. 35%; *p* < 0.001; Table 1; Fig. S8A) but a higher BM blast percentage (3.5% vs. 2.5%; *p* = 0.005; Table 1) than patients in higher ARC cohort. CFU-C assays further confirmed that patients with lower ARC had fewer numbers of CFU-E (30 *vs*. 40/10^5^ BMMNCs; *p* = 0.004; Fig. S8B), BFU-E (12 *vs*. 20/10^5^ BMMNCs; *p* = 0.001; Fig. S8B) and CFU-GM (10 *vs*. 12/10^5^ BMMNCs; *p* = 0.041; Fig. S8B). Moreover, patients with lower ARC more frequently had an EPO level greater than 500 mIU/mL (55.7% vs. 22.9%; *p* < 0.001; Fig. S8C), which may arise from the compensatory EPO secretion from kidney due to the severe ineffective erythropoiesis in lower ARC patients. All these data evidenced that patients with lower ARC had more severe ineffective hematopoiesis, especially erythropoiesis.

**Fig. 3:**
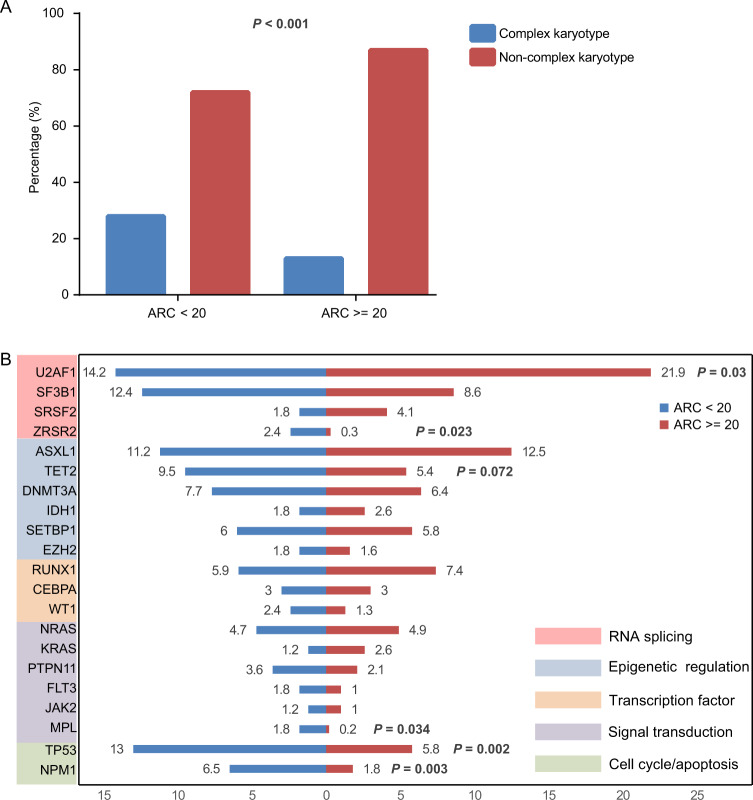
Associations between ineffective erythropoiesis and genetic abnormalities. **a** The percentage of complex karyotype in patients with different degree of ineffective erythropoiesis (stratified by lower and higher ARC). **b** The genetic distributions in MDS patients with lower and higher ARC.

We noticed that patients in the lower ARC cohort who had severe impaired erythropoiesis were more likely to have complex karyotypes compared with patients in the higher ARC cohort (27.5% vs. 12.7%; *p* < 0.001; Fig. 3a). Additionally, we sequenced 112 frequently mutant genes in MDS in diagnostic BM samples from all patients and identified 1470 high-confidence mutations. In total, 643 of 776 patients (82.9%) harbored at least 1 mutation with a median of 2(0–8) mutations per sample. The eight most frequently mutated genes in the total cohort were *U2AF1* (20.2%), *ASXL1* (12.2%), *SF3B1* (9.4%), *TP53* (7.3%), *RUNX1* (7.1%), *DNMT3A* (6.7%), *TET2* (6.3%) and *SETBP1* (5.8%). Distributions of mutations with a variable allele frequency > 2% in the lower and higher ARC cohorts are shown in Fig. 3b. Importantly, *ZRSR2*, *TET2, TP53, NPM1*, and *MPL* were mutated more frequently in patients with lower ARC (*p* = 0.023; *p* = 0.072; *p* = 0.002; *p* = 0.003; *p* = 0.034; Fig. 3b), whereas *U2AF1* mutations were enriched in higher ARC patients (*p* = 0.03; Fig. 3b).

### Severe reduced ARC is a powerful independent prognostic factor of OS in MDS patients

**Fig. 4:**
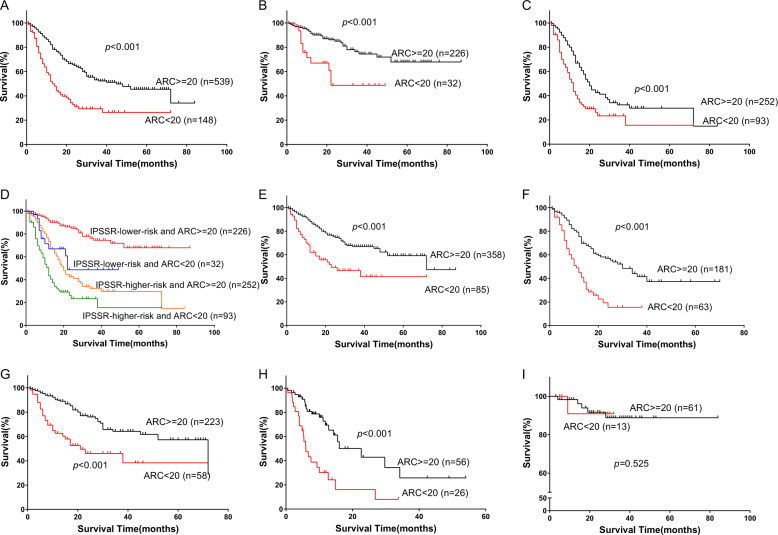
Prognostic impact of ineffective erythropoiesis (assessed by ARC) in MDS patients. **a**–**c** Survival analyses of ARC in the total cohort, IPSS-R lower-risk patients and IPSS-R higher-risk patients, respectively. **d** Survival analyses of MDS patients stratified by IPSS-R risk group and ARC. **e**, **f** Survival analyses of ARC in younger (<60 years) and older (≥60 years) patients. **g**–**i** Survival analyses of ARC in IPSS-R lower-risk patients who received immunosupressive treatment and IPSS-R higher-risk patients who received decitabine therapy or in both categories who received Allo-HSCT.

In total cohort, median OS of patients with lower ARC was 14 months (95% confidence interval [CI], 12–16 months), which was significantly shorter than that of patients with higher ARC (48, 95% CI, 31–65 months; *p* < 0.001; Fig. 4a). In univariate analysis, besides ARC < 20 × 10^9^/L (HR = 2.685, 95% CI, 2.045–3.525, *p* < 0.001), age (≥60 years), IPSS-R higher-risk group and several gene mutations showed significant associations with inferior OS (*p* < 0.05; Table 2).

**Table 2:** Univariate and multivariate analysis of overall survival in the total cohort.

Variables	Univariate analysis			Adjusted by molecular profiles			All-inclusive multivariate analysis		
	HR	95% CI	*p* value	HR	95% CI	*p* value	HR	95% CI	*p* value
Age ≥60 years	1.939	1.493–2.518	<0.001				2.472	1.844–3.315	<0.001
IPSS-R higher-risk group	3.397	2.436–4.736	<0.001				3.047	2.159–4.300	<0.001
ARC < 20 × 10^9^/L	2.685	2.045–3.525	<0.001	2.675	2.021–3.541	<0.001	2.118	1.563–2.870	<0.001
*U2AF1*	1.368	0.995–1.879	0.053	1.407	1.018–1.945	0.039			
*SF3B1*	1.081	0.702–1.664	0.724						
*SRSF2*	1.705	0.953–3.052	0.072	2.229	1.228–4.048	0.008			
*TET2*	1.844	1.217–2.795	0.004	2.102	1.378–3.207	0.001			
*SETBP1*	1.653	1.033–2.647	0.036				2.110	1.200–3.712	0.010
*TP53*	2.915	1.967–4.320	<0.001	2.911	1.937–4.374	<0.001			
*NRAS*	1.837	1.134–2.976	0.014						
*PTPN11*	2.481	1.350–4.559	0.003	1.988	1.058–3.735	0.033	2.206	1.108–4.390	0.024

*HR* hazard ratio, *CI* confidence interval.

Next, to determine the relative contribution of clinical and genetic factors to OS, we generated a multivariable Cox model including variables related to poor prognosis in univariate analysis (*p* < 0.1). We found that ARC < 20 × 10^9^/L retained its significance in OS after adjusting for mutation status and other clinical variables (HR = 2.118, 95% CI, 1.563–2.870, *p* < 0.001). Other independent prognostic variables for poor OS were age (≥60 years), higher-risk group in IPSS-R and mutated *SETBP1* and *PTPN11* (Table 2).

Patients with lower ARC in all IPSS-R risk groups except high-risk group had shorter OS compared with patients with higher ARC (Fig. S9). Analysis of very low-risk group were missing due to all patients in this group had an ARC greater than 20 × 10^9^/L. Both in IPSS-R lower and higher risk cohorts, patients with lower ARC had shorter OS than those with higher ARC (*p* < 0.001; *p* < 0.001; Fig. 4b, c). The median OS of lower-risk patients with lower ARC was 22 months, which was shorter than that of lower-risk patients with higher ARC (not reached; *p* < 0.001; Fig. 4d) and longer than higher-risk patients with lower ARC (12, 95% CI, 10–14 months; *p* = 0.006; Fig. 4d), while was similar to that of higher-risk and higher ARC patients (20, 95% CI, 17–23 months; *p* = 0.370; Fig. 4d).

Given that the BM hematopoietic activity declines with age, we then assessed aging effects by dividing all patients into younger (<60 years) and older (≥60 years) cohorts and analyzed the survival impact of ARC in each age group. As expected, patients with lower ARC had higher proportion of older patients compared with those with higher ARC (43.2% vs. 32.8%; *p* = 0.014; Table 1). Nevertheless, regardless in younger or older cohort, patients with lower ARC had poorer OS than the others (*p* < 0.001; *p* < 0.001; Fig. 4e, f).

### Impact of ARC levels on OS under different treatment regimens

To explore the effect of ARC on survival under different treatment regimens, we compared the OS of patients stratified by ARC who received distinct therapies. We found that among both IPSS-R lower-risk patients, who received cyclosporine (at an initial dose of 3 mg/Kg/day and modified based on blood concentration, hematologic responses and adverse effects) combined with danazol(± thalidomide), and higher-risk patients, who received decitabine therapy at the schedule of 20 mg/m^2^/day for 5 days, every 28 days, patients with lower ARC had shorter OS compared with patients with higher ARC (*p* < 0.001; *p* < 0.001; Fig. 4g, h). Of note, among patients who received Allo-HSCT, no difference of OS was observed between patients with lower and higher ARC (*p* = 0.525; Fig. 4i).

### Impact of different cut-off value of ARC on OS

The above results showed that utilizing ARC of 20 × 10^9^/L as cutoff could properly discriminate prognosis of MDS patients. Meanwhile, we adopted a bio-informatics tool X-tile^28^ to define an ARC threshold of 19.4 × 10^9^/L to predict prognosis and obtained similar results to the cutoff of 20 × 10^9^/L (data were not shown). We also used X-tile to define three subgroups of patients with different outcomes taking 19.4 and 43 × 10^9^/L as threshold. In general, as ARC became lower, the peripheral blood cell count, BM erythroblast percentage and numbers of CFU-C became lower (Table S3, Fig. S10); the incidence of high-risk factors became higher, such as higher BM blast percentage, complex karyotype and several poor prognostic mutations (Table S3 and Figs. S11 and 12). In univariate analysis, patients with ARC < 19.4 × 10^9^/L or 19.4 ≤ ARC < 43 × 10^9^/L both showed poor OS compared with patients with ARC ≥ 43 × 10^9^/L (Table S4 and Fig. S13A). However, no difference of survival was found between patients with 19.4 ≤ ARC < 43 × 10^9^/L and patients with ARC ≥ 43 × 10^9^/L in multivariate analysis or subgroup analysis (Table S4 and Fig. S13B–H). While ARC < 19.4 × 10^9^/L was still an independent poor prognostic factor (Table S4).

## Discussion

In this study, by sorting HSCs and MEPs from MDS patients and inducing differentiation towards erythroid lineage ex vivo, we confirmed the impairments of erythropoietic activity in BM of MDS and, for the first time, found the precocity and premature aging occurring in MDS erythroid cells. To further determine prognostic implications in respect of the severity of ineffective erythropoiesis, we analyzed the clinical data in a large cohort of MDS patients and found that the severe ineffective erythropoiesis (defined by ARC < 20 × 10^9^/L) in MDS served as a powerful independent prognostic factor, which was associated with poor OS and compromised drug response.

The deficiency of erythroid lineage was proved to be most prominent in MDS^4^. Our prior study^13^ showed that numbers of BFU-E and CFU-E in MDS patients were obviously lower than healthy controls, which was confirmed in this study after expanding the sample size. In the present study, we further found that levels of HSCs and MEPs, which is a transitional stage of differentiation to both megakaryocytes and erythrocytes earlier than the BFU-E stage^29,30^, had already reduced in MDS. For a long time, the increased apoptosis of BM erythroid cells was considered to be responsible for ineffective erythropoiesis in MDS. Recently, numerous studies have yielded accumulating evidence about the contribution of pyroptosis, a caspase-1-dependent lytic cell death, and impaired terminal erythroid differentiation in the ineffective erythropoiesis of MDS^8,9,31^. In this study, after inducing the erythroid differentiation from patient-derived MEPs, we unexpectedly found the precocity of erythroid cells during differentiation, namely, the premature generation of hemoglobin at erythroid progenitor cells along with premature aging detected during erythropoiesis. Results of RNA-seq confirmed that aging pathways were up-regulated progressively in MDS patients compared with normal controls, which was consistent with the phenotype discovered by functional experiments. Supposedly, such acceleration of the erythroid differentiation process may intent to compensate for the compromised erythropoiesis, which, however, even worsen erythropoietic disorders; or it may be parallel with other molecular events causing ineffective erythropoiesis from de novo. Definite mechanisms require further investigations.

**Fig. 5:**
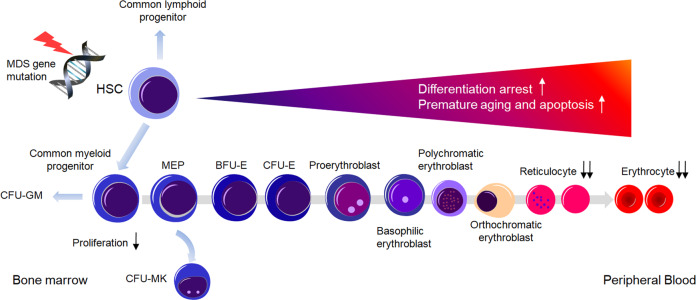
The pattern of ineffective erythropoiesis in MDS patients. A spectrum of somatic mutations occurs in partial HSCs, generating the malignant clone. With the accumulation of genetic lesions in HSPCs during their lifespan, usually accompanied by alterations of BM niche, the disorders of erythropoiesis, including differentiation arrest, precocity and premature aging of erythroid cells and increased apoptosis of erythroid precursors, aggravate gradually and eventually lead to ineffective erythropoiesis and a remarkable decrease in ARC and RBCs in peripheral blood.

All the data mentioned above implied that the defects of erythroid precursors, from both quantitative and qualitative level, contributed to the impairment of erythropoietic activity in MDS. As shown in Fig. 5, a spectrum of somatic mutations occurred in partial HSCs, generating the malignant clone. With the accumulation of genetic lesions in HSPCs during their lifespan, usually accompanied by alterations in BM niche, the disorders of erythropoiesis, including differentiation arrest, precocity and premature aging of erythroid cells and then increased apoptosis of erythroid precursors, aggravate gradually and eventually lead to a remarkable decrease in ARC and RBCs.

Although the ineffective erythropoiesis is one of hallmarks of MDS, there is still no accurate biomarker to quantify it. ARC in peripheral blood is a reliable indicator for reflecting the erythropoietic activity of BM and thus maybe a useful biomarker for evaluating the severity of ineffective erythropoiesis. Using an ARC < 20 × 10^9^/L as the cut-off, which was adopted from the definition criteria of SAA according to BCSH, relationships of ineffective erythropoiesis to survival in MDS were determined. Our data showed that severe ineffective erythropoiesis defined by reduced ARC was an independent prognostic factor of inferior OS in MDS. Several variables should be responsible for this poor outcome. Firstly, the ineffective erythropoiesis is an essential feature of MDS and its degree could reflect the severity of the disease, for patients with more severe dyserythropoiesis may suffer more hits during the process of proliferation and differentiation; secondly, severe ineffective erythropoiesis is closely associated with some poor prognostic factors which has been enrolled in the IPSS-R system, including higher BM blast percentage and more aggressive cytogenetic abnormalities; thirdly, it is correlated with several high-risk gene mutations, such as *TP53* and *ZRSR2*^32–36^.

The immunosuppressive treatment is a fundamental therapy for lower-risk MDS^37^. In recent years, hypo-methylating agents are widely used for higher-risk MDS and significantly improve the prognosis of these patients^38,39^. However, these drugs are not curative therapy for MDS and their effects on survival to some of patients are rather limited. To date, allo-HSCT remains the only treatment choice for a possible cure of MDS^40^. Our primary results showed that the poor outcome of severe reduced ARC could not be reversed by the immunosuppressive or hypo-methylating agents. Also, lower ARC was significantly associated with higher EPO level, which was considered as a biomarker for poor erythroid response to erythropoiesis stimulating agents^41^ Given that the severe ineffective erythropoiesis did not affect survival after HSCT, early HSCT should be suitable therapy for these patients.

There are several limitations to our study. For example, as MDS cells are notoriously difficult to be isolated and cultured ex vivo, although our limited cellular studies enable to provide mechanistic insights of precocity and premature aging regarding ineffective erythropoiesis in MDS, future investigation with larger cohort of patients would warrant thoroughly deciphering its underlying detailed molecular mechanisms.

In conclusion, precocity and premature aging during erythroid differentiation is one of profound defects of erythroid precursors in MDS contributing to the ineffective erythropoiesis. The severe ineffective erythropoiesis defined by ARC < 20 × 10^9^/L is a powerful independent prognostic factor for inferior OS in MDS. Patients with lower ARC could not benefit from immunosuppressive treatment and hypo-methylating agents, wherefore should be candidates for Allo-HSCT.

## Supplementary information

Supplementary information
