# Supplementary material for: Severe ineffective erythropoiesis discriminates prognosis in myelodysplastic syndromes: analysis based on 776 patients from a single centre
Source: Blood Cancer J. 2020 Aug 14;10(8):83. doi: 10.1038/s41408-020-00349-4 (PMC7429953; doi:10.1038/s41408-020-00349-4)
Supplement: Supplementary file 1 — Supplementary information [file 41408_2020_349_MOESM1_ESM.docx]

**Severe ineffective erythropoiesis discriminates prognosis in myelodysplastic syndromes：analysis based on 776 patients from a single centre**

**Supplementary Information**

**Supplementary Methods**

**Cells sorting strategy for megakaryocytic-erythroid progenitors (MEPs)**

***Ex vivo* erythroid differentiation**

**RNA-Seq and Gene sets enrichment analysis (GSEA)**

**Supplementary Figures**

**Supplementary Figure 1. Cell sorting strategy for hematopoietic stem cells (HSCs) (Lin^-^CD34^+^CD38^-^CD45RA^-^CD123^-^) and megakaryocytic-erythroid progenitors (MEPs) (Lin^-^CD34^+^CD38^+^CD45RA^-^CD123^-^).**

**Supplementary Figue 2. Quantitative analysis of HSCs, MEPs and erythroid progenitors from MDS patients and healthy donors (HD).**

**Supplementary Figure 3. Hemoglobin synthesis analysis and senescent cells counting of erythroid cells derived from MDS patients and HD.**

**Supplementary Figure 4. HALLMARK gene sets enrichment analysis between MDS patients and HD at different differentiation stages.**

**Supplementary Figure 5. Aging-associated genes were induced in MDS patient cells compared with HD.**

**Supplementary Figure 6. Downregulation of *ERCC1* expression could inhibit aging process and promote proliferation of K562 cells.**

**Supplementary Figure 7. Distributions of absolute reticulocyte counts (ARC), which was shown by mean and standard deviation, among different WHO diagnostic categories and IPSS-R risk groups.**

**Supplementary Figure 8. The percentage of bone marrow (BM) erythroblasts, numbers of colony-forming unit cell (CFU-C) and distributions of erythropoietin (EPO) levels between patients with ARC<20×10^9^/L and ≥20×10^9^/L.**

**Supplementary Figure 9. Overall survival of patients with ARC<20×10^9^/L and ≥20×10^9^/L in different IPSS-R risk groups.**

**Supplementary Figure 10. The percentage of BM erythroblasts, numbers of CFU-C and distributions of EPO levels among three subgroups of patients according to ARC.**

**Supplementary Figure 11. The percentage of complex karyotype among three subgroups.**

**Supplementary Figure 12. Mutation topography of patients among three groups.**

**Supplementary Figure 13. Prognostic impact of different ARC levels in MDS patients.**

**Supplementary Tables**

**Supplementary table 1. Clinical characteristics of 31 MDS patients enrolled in the functional studies and RNA-Seq analysis.**

**Supplementary table 2. The primers used for real-time PCR or the short hairpin RNA sequences.**

**Supplementary table 3. Comparisons of clinical and laboratory characteristics of MDS patients grouped by ARC of 19.4 and 43×10^9^/L.**

**Supplementary table 4. Univariate and multivariate analysis of overall survival in the total cohort (using ARC of 19.4 and 43×10^9^/L as cutoff).**

**Supplementary Methods**

**Cells sorting strategy for megakaryocytic-erythroid progenitors (MEPs)**

Cells were stained with a lineage-marker cocktail (biotinylated anti-CD2, -CD3, -CD11b, -CD14, -CD15, -CD16, -CD19, -CD56, -CD123, and -CD235a) and subsequently magnetically labeled with Anti-Biotin MicroBeads (MiltenyiBiotec, Cat#130-092-211). For each assay, 1 × 10^6^Lin^-^cells were stained with APC-H7-conjugated anti-CD45RA (BD Biosciences, Cat# 560674), PerCP-Cy5.5-conjugated anti-CD123 (BD Biosciences, Cat# 560904), PE-Cy7-conjugated anti-CD38 (BD Biosciences, Cat#335790) and APC-conjugated anti-CD34 (BD Biosciences, Cat#555824) antibody at 4 °C for 30 minutes in the dark. HSCs (CD34+CD38-CD45RA-CD123-) and MEPs (CD34+CD38+CD45RA-CD123-) were sorted with an Aria III instrument (BD Biosciences). Data were analyzed with FlowJo (version 7.6.1).

***Ex vivo* erythroid differentiation**

The purified CD34^+^ cells or FACS sorted BM MEPs were cultured in the basic Iscove’s modified Dulbecco’s medium (IMDM) (Sigma, Cat# 13390) supplemented with inositol (Sigma, Cat#I5125), 20% BIT (v/v) (StemCell Technologies, Cat# 9500), monothioglycerol (Sigma, Cat# M6145), folic acid (Sigma, Cat# F7876), ferrous sulfate (Sigma, Cat# F8633), ferrous nitrate (Sigma, Cat# 8508), glutamine (Gibco, Cat# 25030) and 1% penicillin and streptomycin (v/v). From day 0 to 8, recombinant human stem cell factor (SCF)(Prospec, Cat# CYT-255), recombinant human interleukin-3 (IL-3) (Sigma, Cat# I1646), recombinant human erythropoetinalfa (EPO) (ProTech, Cat# 100-64) and hydrocortisone (Sigma, Cat# H2270) was supplemented to the culture medium. From day 8 through 14, SCF and EPO were included in the culture medium, and only EPO was included during day 14 to 18.

**RNA-Seq and Gene sets enrichment analysis (GSEA)**

Rawdata of RNA-seq were aligned to the human genome using HISAT2(1). The genes was annotated by GENCODE version 29 (<https://www.gencodegenes.org/>). To distinguish the first-strand-specific cDNA library, the parameter (--rna-strandness RF) was applied in HISAT2. Aligned reads were used to quantify gene expression by StringTie(1) with default parameters. Gene sets “GO_Aging” and “GO_Apoptotic_Signaling_Pathway” were downloaded from <http://software.broadinstitute.org/gsea/msigdb/genesets.jsp.> HALLMARK gene sets(2) were derived from MSigDB. *p* values < 0.05 were considered significant in GSEA analysis.

**References:**

1. Pertea, M., Kim, D., Pertea, G.M., Leek, J.T. & Salzberg, S.L. Transcript-level expression analysis of RNA-seq experiments with HISAT, StringTie and Ballgown. Nat Protoc**11**, 1650-1667 (2016).

2. Liberzon, A. et al. The Molecular Signatures Database (MSigDB) hallmark gene set collection. Cell Syst **1**, 417-425 (2015).

**Supplementary Figure 1. Cell sorting strategy for hematopoietic stem cells (HSCs) (Lin^-^CD34^+^CD38^-^CD45RA^-^CD123^-^) and megakaryocytic-erythroid progenitors (MEPs) (Lin^-^CD34^+^CD38^+^CD45RA^-^CD123^-^).**

**Supplementary Figue 2. Quantitative analysis of HSCs, MEPs and erythroid progenitors from MDS patients and healthy donors (HD).** (A-B) The percentage of HSCs (marked with Lin^-^CD34^+^CD38^-^CD45RA^-^CD123^-^) and MEPs (marked with Lin^-^CD34^+^CD38^+^CD45RA^-^CD123^-^) in Lin- cells derived from MDS patients and HD. (C) CFU-C assays were performed using the MethoCultTM H4435 Enriched (STEMCELL Technologies, Vancouver, Canada.). Numbers of BFU-E and CFU-E of MDS patients were significantly lower than that of HD.

**
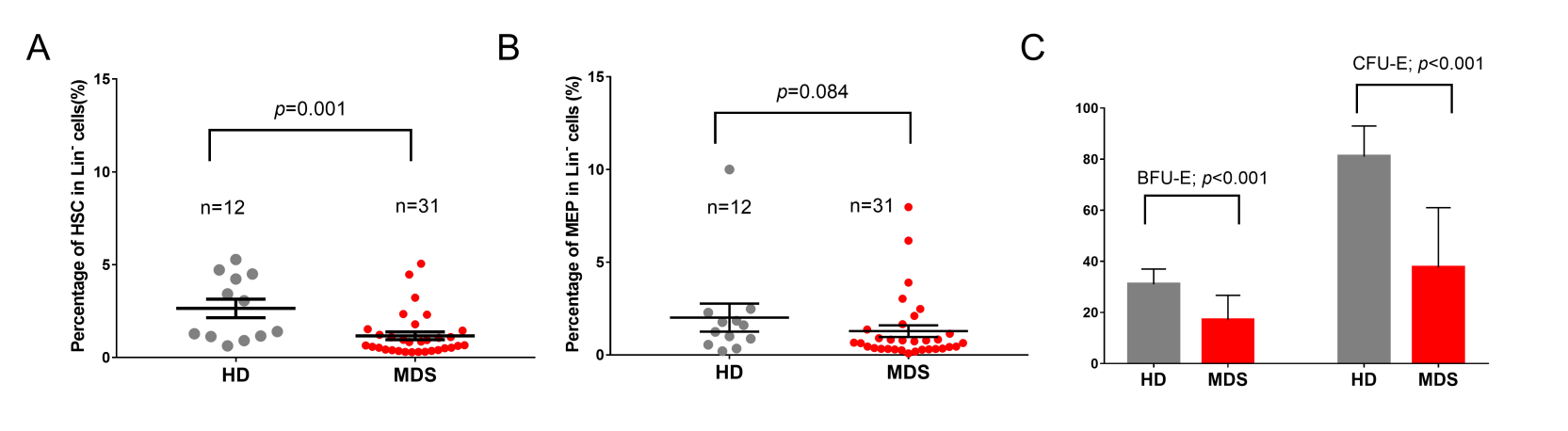
**

**Supplementary Figure 3. Hemoglobin synthesis analysis and senescent cells counting of erythroid cells derived from MDS patients and HD.** (A) Representative images of Benzidine stained erythroid cells cultured for different days as indicated. (B) The percentage of benzidine reactive cells in total erythroid cells. (C) The percentage of β-galactosidase staining positive cells in total erythroid cells.

**
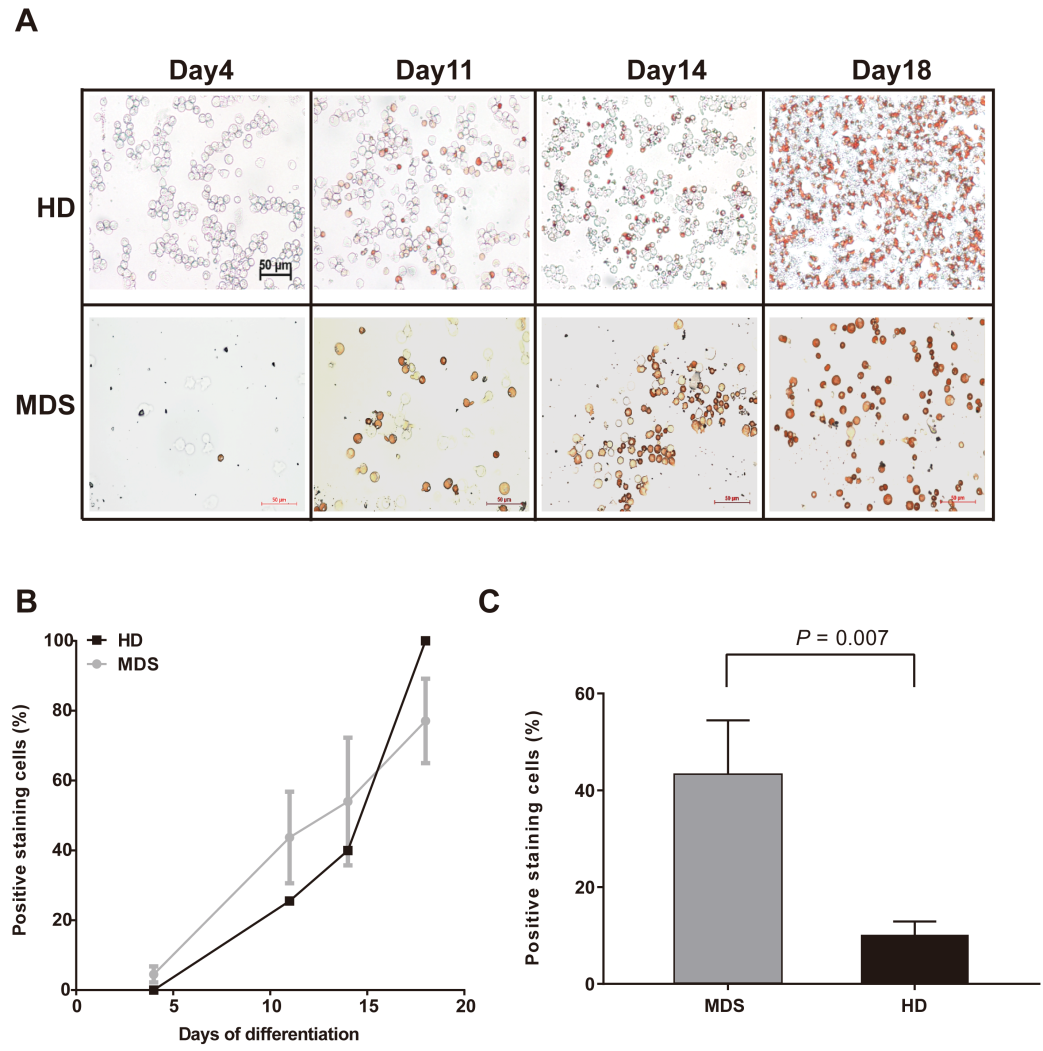
**

**Supplementary Figure 4. HALLMARK gene sets enrichment analysis between MDS patients and HD at different differentiation stages.** The red color indicated highly expressing terms in MDS, and the blue color indicated highly expression terms in HD. Significantly terms (Nominal *p* value < 0.05) are highlighted through different panes.

**
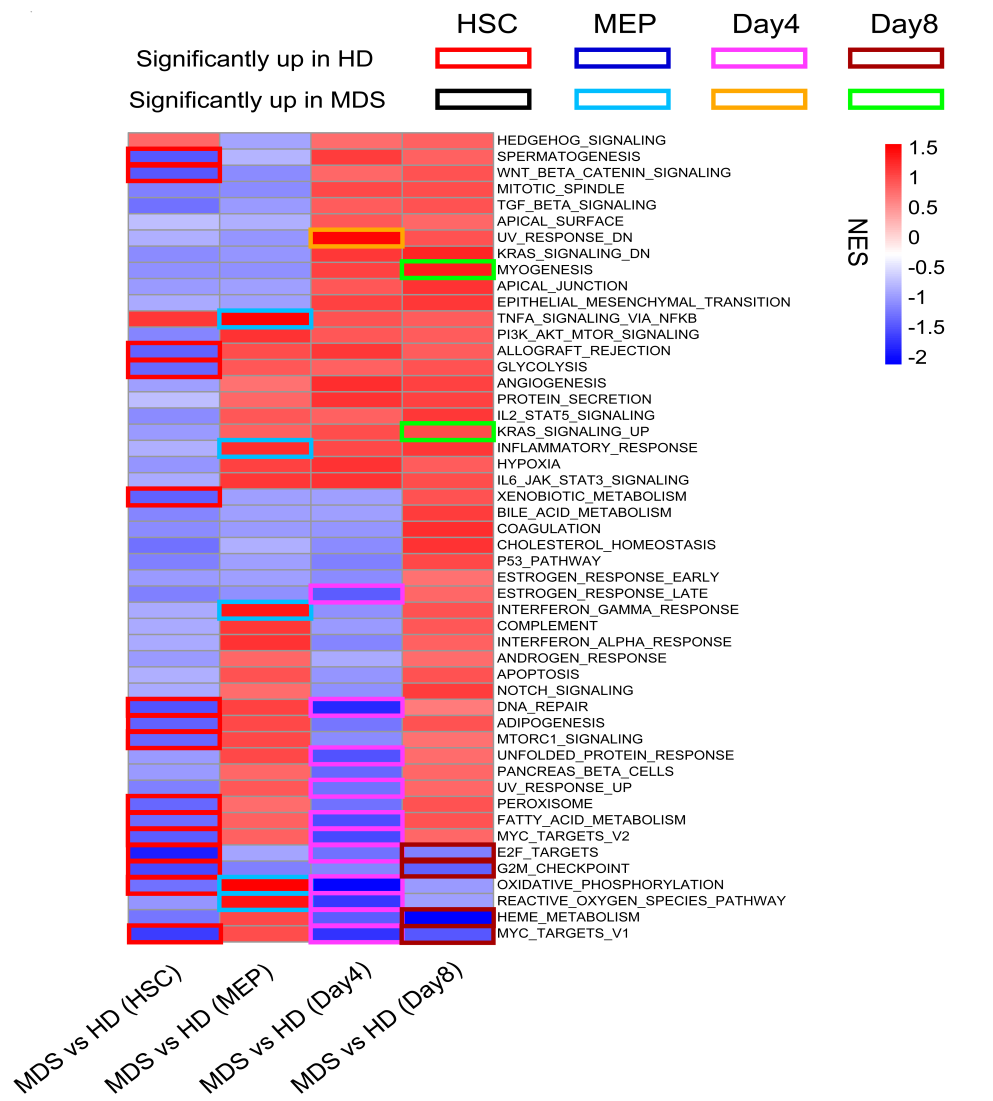
**

**Supplementary Figure 5. Aging-associated genes were induced in MDS patient cells compared with HD.**

**
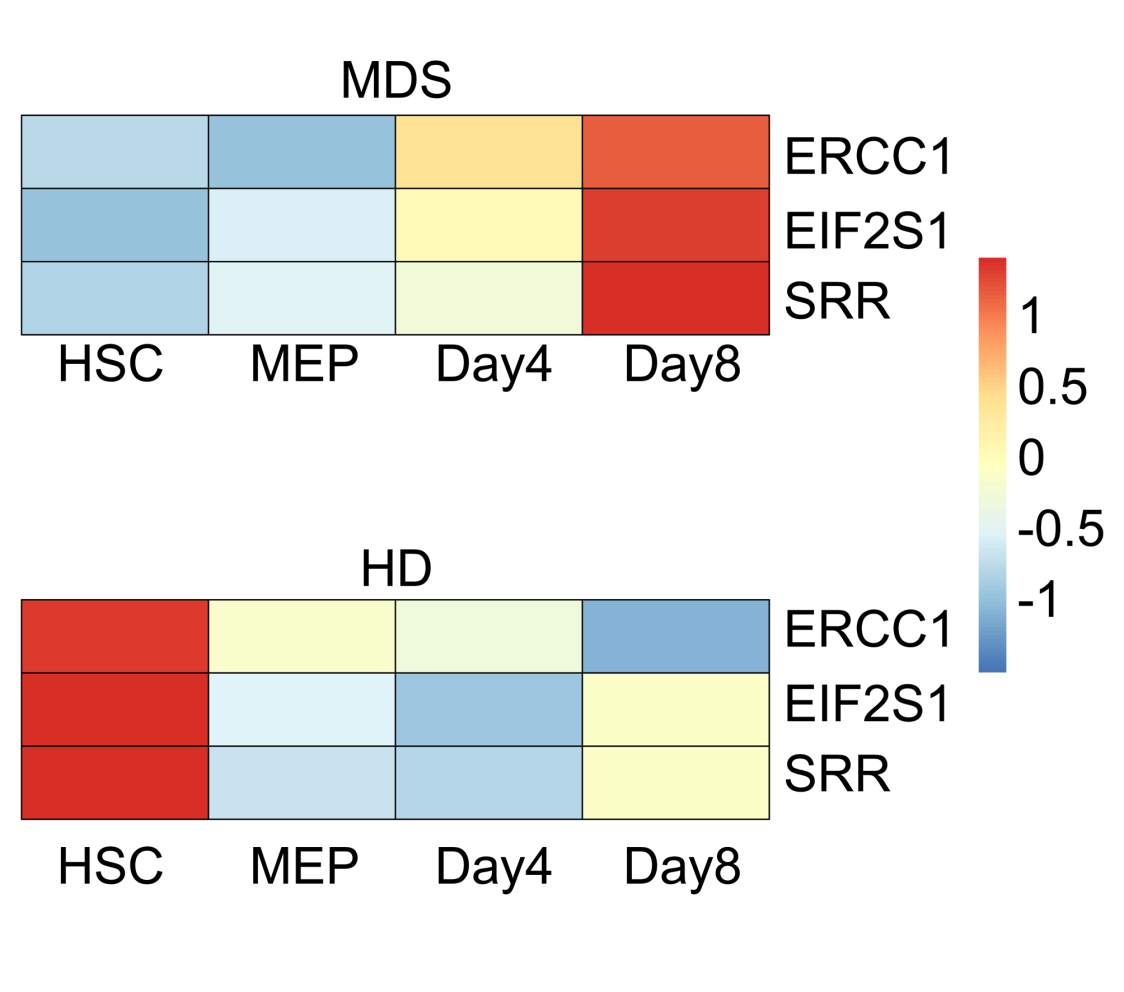
**

**Supplementary Figure 6. Downregulation of *ERCC1* expression could inhibit aging process and promote proliferation of K562 cells.** (A) qRT-PCR analysis and WB confirmed the expression of *ERCC1* in K562 cells were significantly reduced after infection of *ERCC1-*targeting shRNAs*.* (B) Representative β-galactosidase staining images in shRNA lentivirus infected K562 cells or scramble counterparts (Scale bar=20μm) at day3 of hemin-induced erythroid differentiation. (C) Quantitative analysis confirmed that K562 cells were less frequently to be dyed blue after knocking down *ERCC1*. (D) Cell counting suggested that fold change of proliferation of K562 cells from day0 to day3 of hemin-induced differentiation were increased after knocking down *ERCC1.*


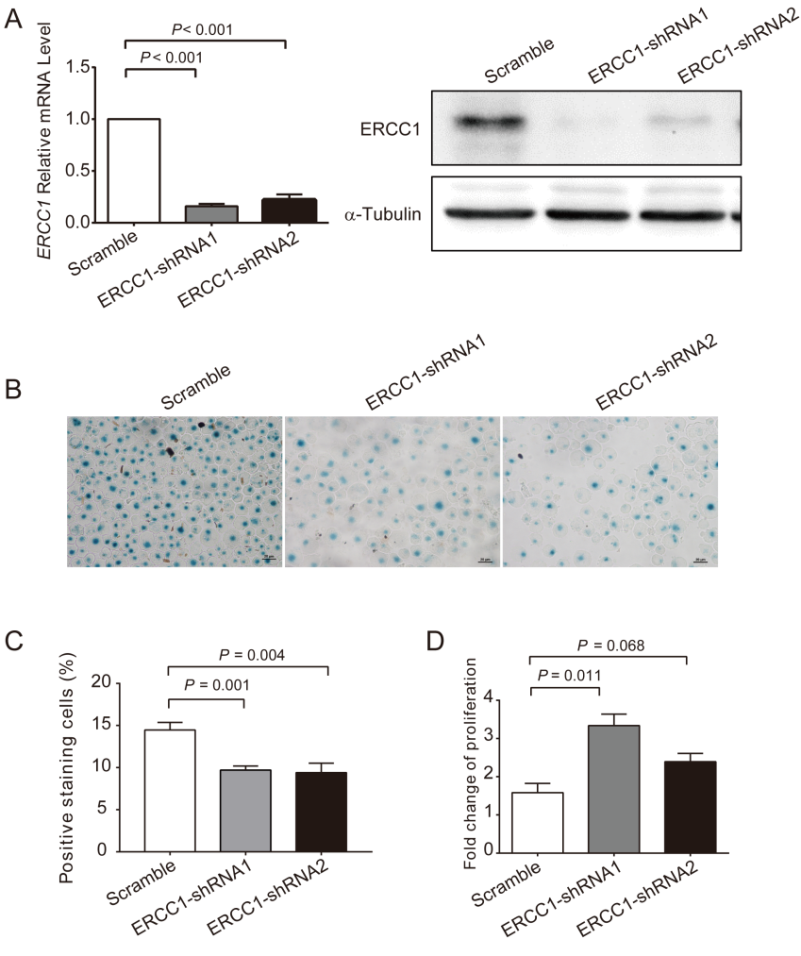


**Supplementary Figure 7. Distributions of absolute reticulocyte counts (ARC), which was shown by mean and standard deviation, among different WHO diagnostic categories and IPSS-R risk groups.** (A) No significant difference was found in levels of ARC between patients in different WHO diagnostic categories. (B) Patients in higher IPSS-R risk groups had lower levels of ARC.

**
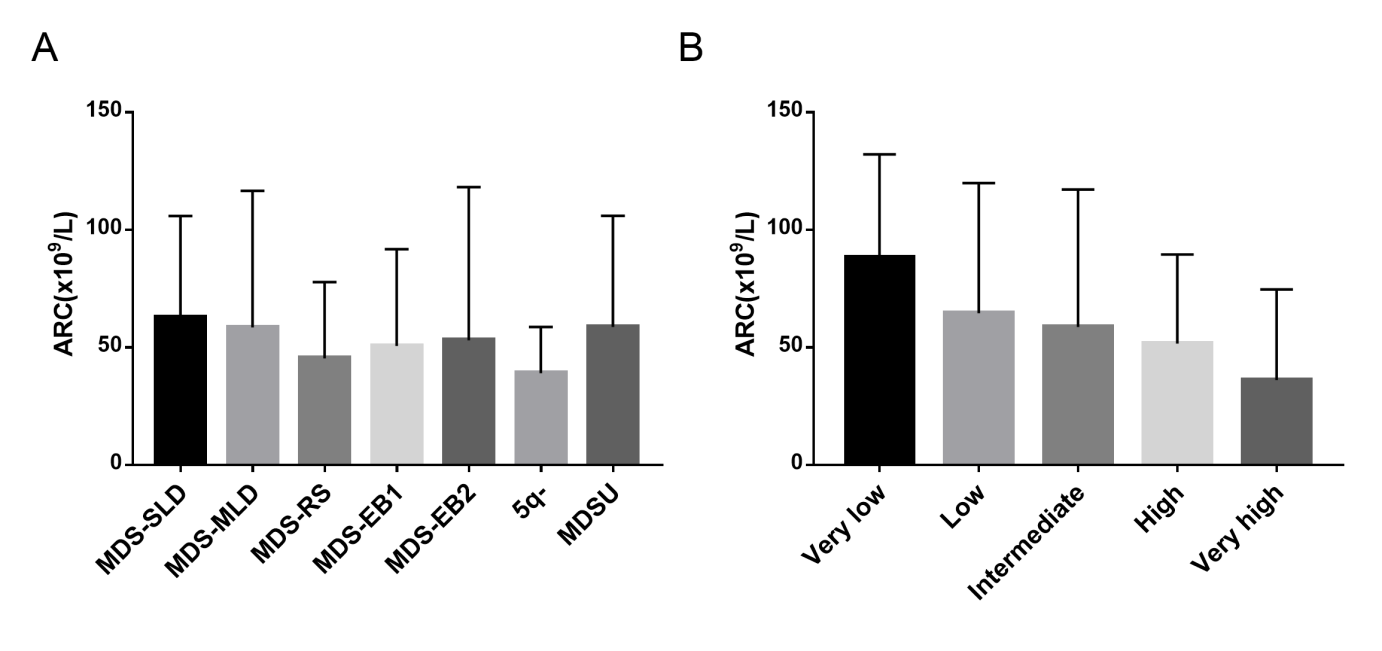
**

**Supplementary Figure 8. The percentage of bone marrow (BM) erythroblasts, numbers of colony-forming unit cell (CFU-C) and distributions of erythropoietin (EPO) levels between patients with ARC<20×10^9^/L and ≥20×10^9^/L.** (A) MDS patients with ARC fewer than 20×10^9^/L had lower percent of bone marrow erythroid precursor cells. (B) Also, patients with ARC fewer than 20×10^9^/L had lower numbers of hematopoietic progenitors, including CFU-E, BFU-E and CFU-GM. (C) Patients with ARC fewer than 20×10^9^/L had higher proportion of patients with erythropoietin (EPO) level greater than 500mIU/mL.

**
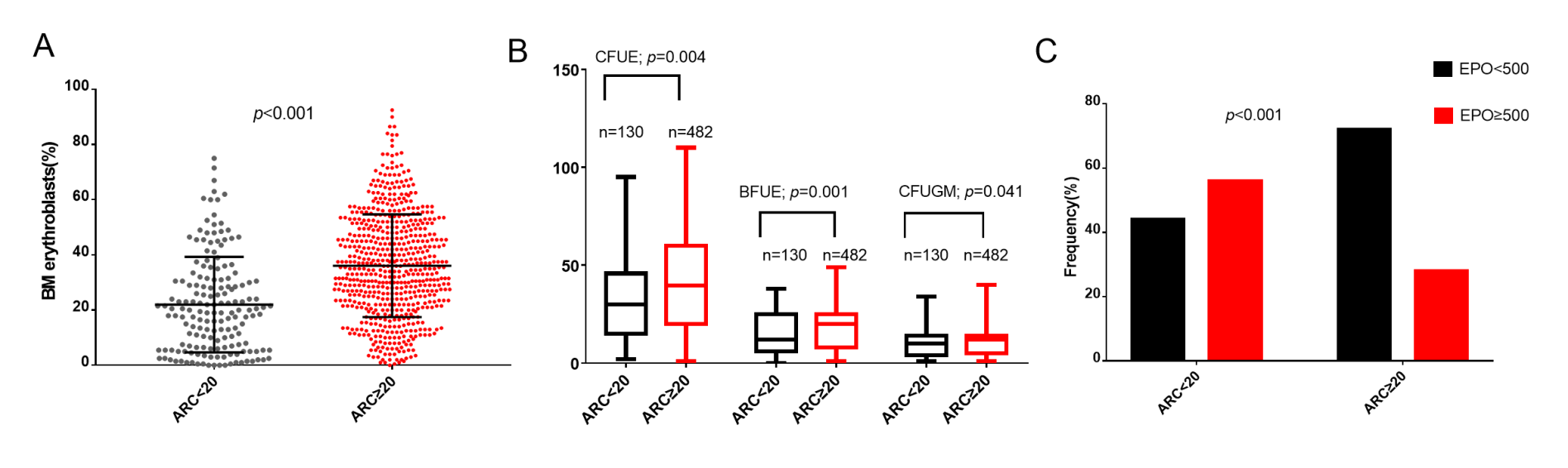
**

**Supplementary Figure 9. Overall survival of patients with ARC<20×10^9^/L and ≥20×10^9^/L in different IPSS-R risk groups.** Data in IPSS-R very low-risk group were missing because all patients in this group had an ARC greater than 20×10^9^/L.


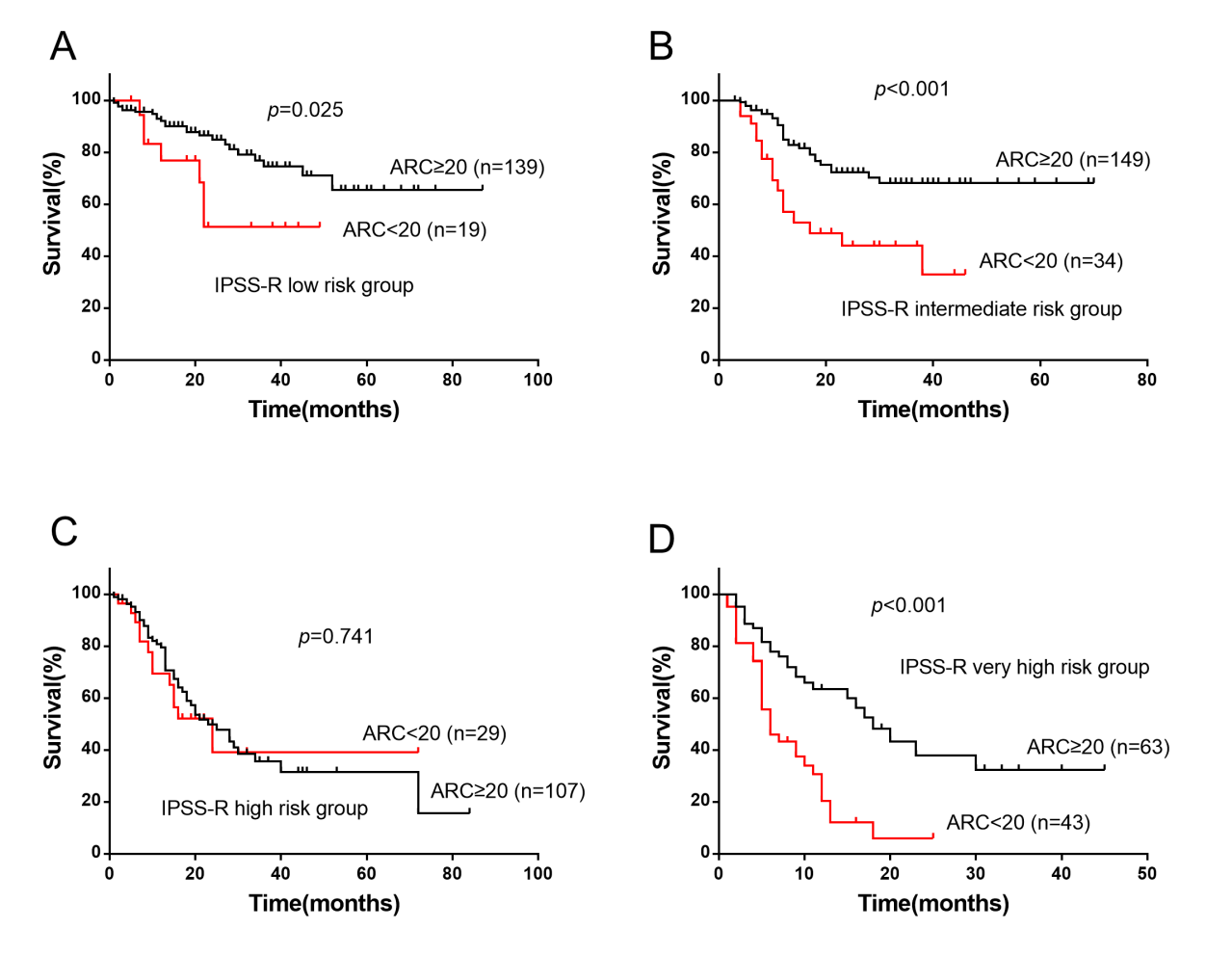


**Supplementary Figure 10. The percentage of BM erythroblasts, numbers of CFU-C and distributions of EPO levels among three subgroups of patients according to ARC.**


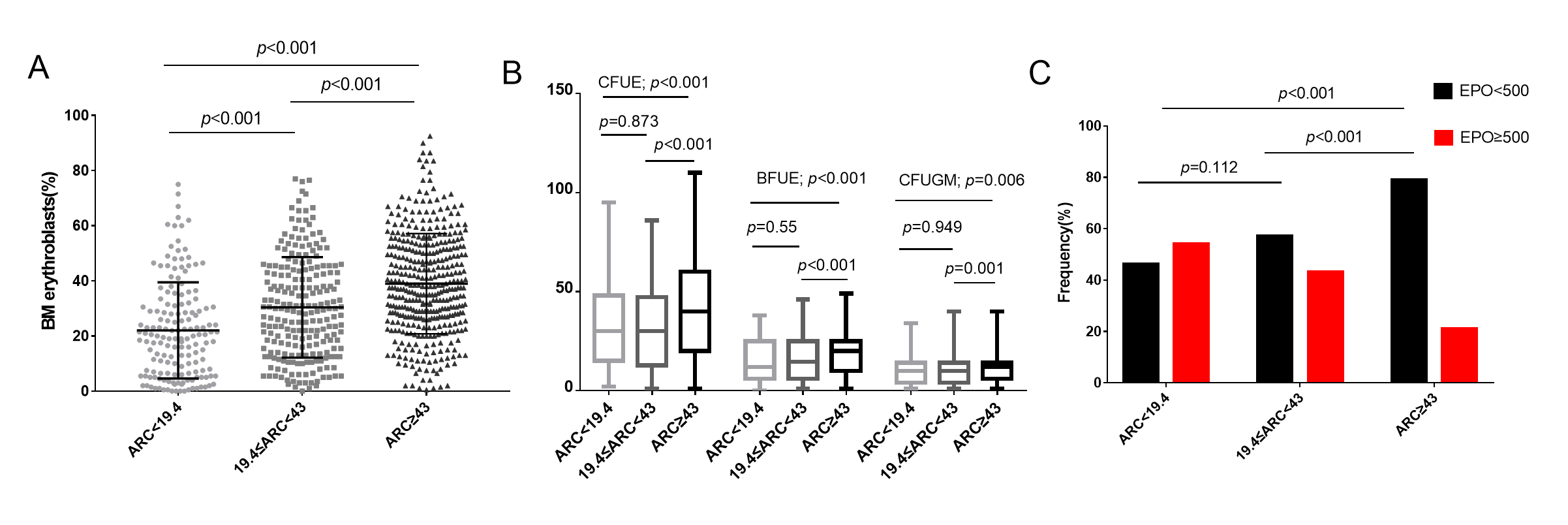


**Supplementary Figure 11. The percentage of complex karyotype among three subgroups.** ARC<19.4×10^9^/L *vs*. 19.4≤ARC<43×10^9^/L, P=0.142; ARC<19.4×10^9^/L *vs.* ARC≥43×10^9^/L, P<0.001; 19.4≤ARC<43×10^9^/L *vs.* ARC≥43×10^9^/L, P<0.001.

**Supplementary Figure 12. Mutation topography of patients among three groups.**

**Supplementary Figure 13. Prognostic impact of different ARC levels in MDS patients.** P1: ARC<19.4×10^9^/L *vs*. 19.4≤ARC<43×10^9^/L; P2: ARC<19.4×10^9^/L *vs.* ARC≥43×10^9^/L; P3: 19.4≤ARC<43×10^9^/L *vs.* ARC≥43×10^9^/L.

**
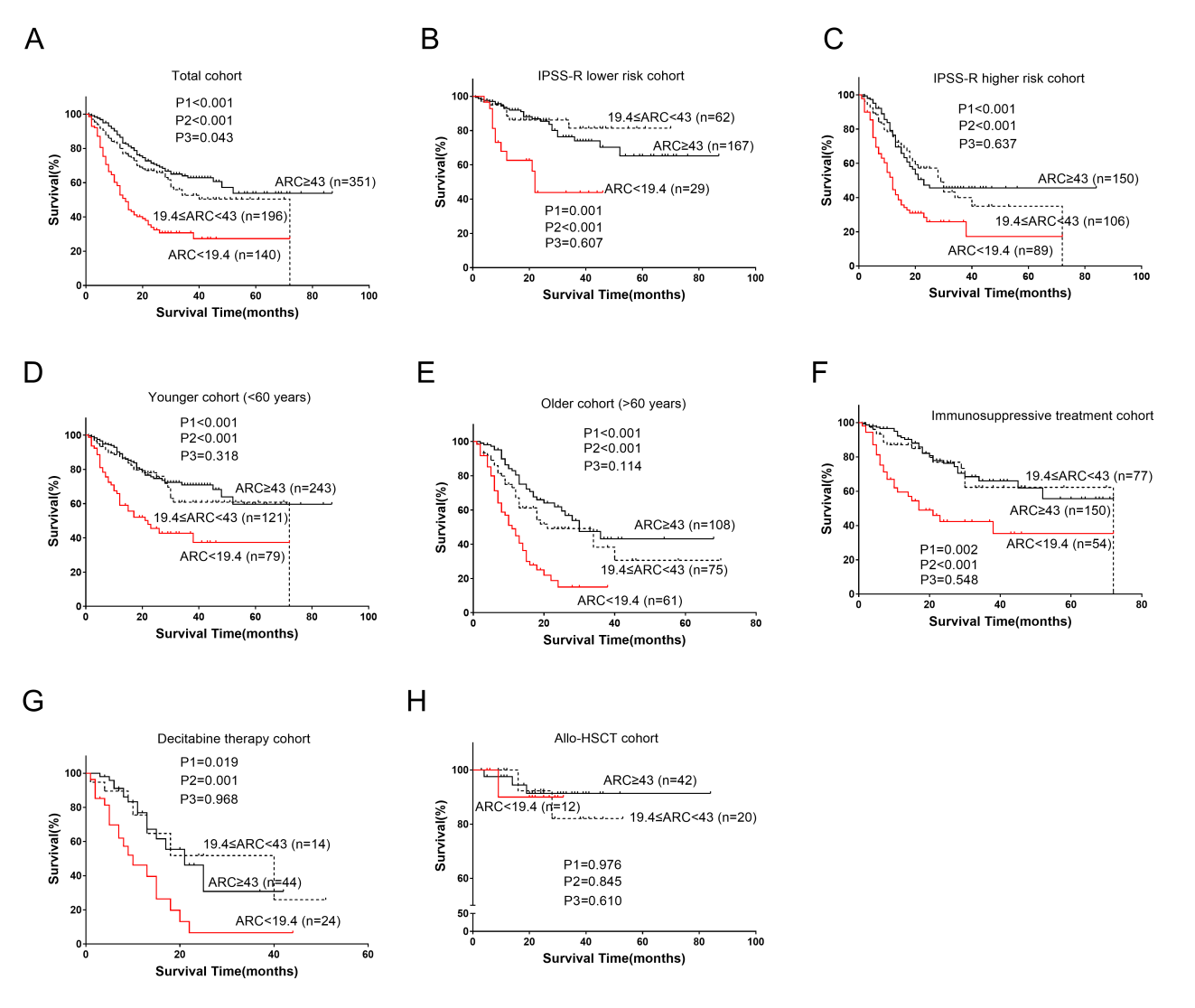
**

**Supplementary Table 1. Clinical characteristics of 31 MDS patients enrolled in the functional studies and RNA-Seq analysis**

| No.of pts | Age | Sex | Diagnosis WHO(2016) | IPSS-R | ARC(×10^9^/L) | Hb(g/L) | ANC(×10^9^/L) | PLT(×10^9^/L) | Cytogenetics |
| --- | --- | --- | --- | --- | --- | --- | --- | --- | --- |
| 01 | 42 | F | MDS-MLD | Very low | 27.4 | 108 | 0.87 | 168 | Normal |
| 02 | 54 | M | MDS-EB1 | Very high | 39 | 110 | 0.28 | 58 | 59～60,XY,+1,+2,+6,-8,+9,+13,+14,+15,-17,-18,+19,?del(20)(q12),+21,+22,+4～6mar,inc[cp13] |
| 03 | 67 | M | MDS-EB2 | Very high | 14.7 | 67 | 1.1 | 77 | 46,XY,t(6;9)(p23;q34)[4]/47,XY,+?8[3]/46,XY[13] |
| 04 | 61 | M | MDS-EB1 | / | 33.8 | 75 | 0.85 | 38 | NA |
| 05 | 75 | M | MDS-MLD | Low | 75.7 | 112 | 0.85 | 84 | Normal |
| 06 | 51 | F | MDS-MLD | / | 11.6 | 77 | 0.57 | 131 | NA |
| 07 | 53 | M | MDS-MLD-RS | Low | 30.4 | 62 | 2.21 | 82 | Normal |
| 08 | 65 | M | MDS-MLD | Low | 37.7 | 86 | 1.09 | 25 | Normal |
| 09 | 49 | F | MDS-MLD | Low | 22.5 | 97 | 1.59 | 225 | 48,XX,+8,+9[3]/46,XX[6] |
| 10 | 48 | F | MDS-MLD | Low | 111.4 | 123 | 1.3 | 51 | 47,XX,+8[8]/46,XX[5] |
| 11 | 37 | M | MDS-EB2 | / | 56.6 | 65 | 1.02 | 10 | NA |
| 12 | 66 | M | MDS-SLD | Inter | 10.6 | 71 | 0.64 | 2 | Normal |
| 13 | 66 | M | MDS-EB1 | Inter | 90.7 | 104 | 0.79 | 134 | Normal |
| 14 | 44 | M | MDS-MLD | Inter | 35.2 | 68 | 1 | 19 | 46,XY,del(20)(q12)[20] |
| 15 | 33 | F | MDS-EB1 | Inter | 42.2 | 65 | 0.94 | 134 | Normal |
| 16 | 36 | M | MDS-SLD | Inter | 50 | 59 | 1.2 | 20 | Normal |
| 17 | 46 | M | MDS-MLD | Inter | 147.7 | 91 | 1.83 | 66 | 47,XY,+8[11]/46,XY[9] |
| 18 | 37 | F | MDS-MLD | Inter | 44.8 | 59 | 0.6 | 57 | 46,XY,del(20)(q12)[20] |
| 19 | 48 | M | MDS-SLD-RS | Inter | 170.7 | 64 | 0.39 | 55 | Normal |
| 20 | 51 | M | MDS-MLD | / | 85.5 | 116 | 7.14 | 77 | NA |
| 21 | 32 | M | MDS-MLD | Inter | 47.7 | 75 | 3.23 | 111 | 47,XY,dup(1)(q21q32),+8[3]/46,XY,dup(1)(q21q32)[13]/46,XY[4] |
| 22 | 14 | F | MDS-MLD | Inter | 35.6 | 60 | 2.27 | 12 | Normal |
| 23 | 14 | F | MDS-EB1 | High | 103.8 | 75 | 0.66 | 122 | Normal |
| 24 | 60 | M | MDS-EB2 | High | 9.3 | 74 | 0.35 | 68 | Normal |
| 25 | 67 | F | MDS-MLD | High | 50.4 | 90 | 1.24 | 35 | 47,XX,del(3)(p21),del(5)(q13q33),+22[18]/46,XX[2] |
| 26 | 39 | M | MDS-EB2 | High | 43.8 | 97 | 0.52 | 98 | Normal |
| 27 | 75 | F | MDS-EB1 | High | 3.7 | 69 | 0.6 | 109 | 46,XX,14ps+[20] |
| 28 | 42 | F | MDS-MLD | High | 29.9 | 67 | 0.98 | 95 | 47,XX,+?8,?der(11),inc[cp20] |
| 29 | 63 | M | MDS-EB1 | High | 183.5 | 92 | 0.28 | 53 | Normal |
| 30 | 52 | M | MDS-EB2 | High | 214.8 | 93 | 1.8 | 10 | Normal |
| 31 | 64 | F | MDS-EB1 | / | 41.7 | 73 | 1.6 | 21 | NA |

NA, Not available

**Supplementary Table 2. The primers used for real-time PCR or the short hairpin RNA sequences.**

| Primer | Sequence 5'-3' |
| --- | --- |
| ACTIN-F | CTCTTCCAGCCTTCCTTCCT |
| ACTIN-R | AGCACTGTGTGTTGGCGTACAG |
| ERCC1-F | TTTGGCGACGTAATTCCCGA |
| ERCC1-R | GCCCATGGATGTAGTCTGGG |
| ERCC1 sh1 | GCCAAGCCCTTATTCCGATCTA |
| ERCC1 sh2 | GCAAGAGAAGATCTGGCCTTAT |

**Supplementary Table 3. Comparisons of clinical and laboratory characteristics of MDS patients grouped by ARC of 19.4 and 43×10^9^/L.**

| **Characteristics** | **ARC<19.4×10^9^/L(N=161)** | **19.4≤ARC<43×10^9^/L(N=223)** | **ARC≥43×10^9^/L(N=392)** | **P1** | **P2** | **P3** |
| --- | --- | --- | --- | --- | --- | --- |
| Sex,n(%) |  |  |  | 0.191 | 0.042 | 0.546 |
| Male | 113(70.2%) | 142(63.7%) | 239(61.0%) |  |  |  |
| Female | 48(29.8%) | 81(36.3%) | 153(39.0%) |  |  |  |
| Age,median(range),y | 57(16-83) | 55(15-83) | 52(14-83) | 0.178 | 0.005 | 0.109 |
| Age≥60y,n(%) | 71(44.1%) | 81(36.3%) | 120(30.6%) | 0.139 | 0.003 | 0.153 |
| WHO classification 2016,n(%) |  |  |  | 0.299 | 0.034 | 0.317 |
| MDS-SLD | 4(2.5%) | 12(5.4%) | 18(4.6%) |  |  |  |
| MDS-RS-SLD | 4(2.5%) | 9(4.0%) | 9(2.3%) |  |  |  |
| MDS-MLD | 67(41.6%) | 101(45.3%) | 210(53.6%) |  |  |  |
| MDS-RS-MLD | 5(3.1%) | 3(1.3%) | 7(1.8%) |  |  |  |
| MDS-EB1 | 38(23.6%) | 44(19.7%) | 71(18.1%) |  |  |  |
| MDS-EB2 | 40(24.8%) | 45(20.2%) | 60(15.3%) |  |  |  |
| MDS with isolated del(5q) | 0 | 4(1.8%) | 3(0.8%) |  |  |  |
| MDS-U | 3(1.9%) | 5(2.2%) | 14(3.6%) |  |  |  |
| Hb,median(range),g/L | 64(31-138) | 73(39-146) | 90(38-155) | <0.001 | <0.001 | <0.001 |
| WBC,median(range),×10^9^/L | 2.51(0.71-21.17) | 2.67(0.62-15.74) | 2.87(0.61-20.42) | 0.175 | 0.003 | 0.086 |
| ANC,median(range),×10^9^/L | 1.01(0.04-13.22) | 1.15(0-10.99) | 1.21(0-17.37) | 0.129 | 0.003 | 0.136 |
| PLT,median(range),×10^9^/L | 51(2-536) | 59(5-694) | 66(2-607) | 0.012 | 0.024 | 0.549 |
| BM erythroblasts,median(range),% | 19.75(0-75) | 29.25(0-77) | 37.5(0-92.5) | <0.001 | <0.001 | <0.001 |
| Sum of proerythroblast and basophilic erythroblast(E1),median(range),% | 1(0-30) | 1(0-17.5) | 2(0-18.5) | 0.728 | 0.001 | 0.001 |
| Sum of polychromatic and orthochromatic erythroblast(E2), median(range),% | 17.5(0-67) | 27(0-73) | 35.5(0.5-88) | <0.001 | <0.001 | <0.001 |
| Ratio of E1 to E2,median(range) | 0.057(0-1.09) | 0.04(0-1.75) | 0.05(0-2) | 0.098 | 0.344 | 0.164 |
| BM blast,median(range),% | 3.5(0-19.5) | 2.5(0-19.5) | 2.0(0-19.0) | 0.087 | 0.001 | 0.119 |
| IPSS-R karyotype,n(%),N=678 |  |  |  | 0.187 | <0.001 | 0.002 |
| Very good | 1(0.7%) | 4(2.1%) | 3(0.8%) |  |  |  |
| Good | 64(47.4%) | 105(55.3%) | 213(60.3%) |  |  |  |
| Intermediate | 26(19.3%) | 41(21.6%) | 97(27.5%) |  |  |  |
| Poor | 10(7.4%) | 10(5.3%) | 20(5.7%) |  |  |  |
| Very poor | 34(25.2%) | 30(15.8%) | 20(5.7%) |  |  |  |
| Complex karyotype,n(%) | 37(27.4%) | 38(20.0%) | 32(9.1%) | 0.142 | <0.001 | <0.001 |
| IPSS-R risk group,n(%),N=678 |  |  |  | 0.012 | <0.001 | <0.001 |
| Very low | 0 | 1(0.5%) | 20(5.7%) |  |  |  |
| Low | 18(13.3%) | 45(23.7%) | 106(30.0%) |  |  |  |
| Intermediate | 40(29.6%) | 54(28.4%) | 122(34.6%) |  |  |  |
| High | 29(21.5%) | 50(26.3%) | 73(20.7%) |  |  |  |
| Very high | 48(35.6%) | 40(21.1%) | 32(9.1%) |  |  |  |
| IPSS-R two groups,n(%) |  |  |  | 0.149 | <0.001 | <0.001 |
| Lower-risk | 37(27.4%) | 67(35.3%) | 181(51.3%) |  |  |  |
| Higher-risk | 98(72.6%) | 123(64.7%) | 172(48.7%) |  |  |  |

MDS, myelodysplastic syndrome; ARC, absolute reticulocyte count; MDS-SLD, MDS with single lineage dysplasia; MDS-RS-SLD, MDS with ring sideroblasts with single lineage dysplasia; MDS-MLD, MDS with multilineage dysplasia; MDS-RS-MLD, MDS with ring sideroblasts with multilineage dysplasia; MDS-EB1, MDS with excess blasts-1; MDS-EB2, MDS with excess blasts-2; MDS-U, MDS unclassifiable; Hb, haemoglobin; WBC, white blood count; ANC, absolute neutrophil count; PLT, platelet count; BM, bone marrow; IPSS-R, Revised International Prognostic Scoring System. P1: ARC<19.4×10^9^/L *vs*. 19.4≤ARC<43×10^9^/L; P2: ARC<19.4×10^9^/L *vs.* ARC≥43×10^9^/L; P3: 19.4≤ARC<43×10^9^/L *vs.* ARC≥43×10^9^/L.

**Supplementary table 4. Univariate and multivariate analysis of overall survival in the total cohort (using ARC of 19.4 and 43×10^9^/L as cutoff).**

|  | Univariate analysis | | | |  | | Adjusted by molecular profiles | | |  | | All-inclusive multivariate analysis | | |
| --- | --- | --- | --- | --- | --- | --- | --- | --- | --- | --- | --- | --- | --- | --- |
| Variables | | HR | 95% CI | *p* value | |  | HR | 95% CI | *p* value | |  | HR | 95% CI | *p* value |
| Age≥60 years | | 1.939 | 1.493-2.518 | <0.001 | |  |  |  |  | |  | 2.491 | 1.854-3.347 | <0.001 |
| IPSS-R higher-risk group | | 3.397 | 2.436-4.736 | <0.001 | |  |  |  |  | |  | 3.015 | 2.131-4.264 | <0.001 |
| ARC<19.4 *vs.* ≥43×10^9^/L | | 3.337 | 2.455-4.535 | <0.001 | |  | 3.289 | 2.398-4.512 | <0.001 | |  | 2.181 | 1.552-3.065 | <0.001 |
| 19.4≤ARC<43 *vs.* ≥43×10^9^/L | | 1.394 | 1.004-1.936 | 0.043 | |  | 1.474 | 1.058-2.054 | 0.022 | |  | 1.037 | 0.722-1.488 | 0.845 |
| *U2AF1* | | 1.368 | 0.995-1.879 | 0.053 | |  |  |  |  | |  |  |  |  |
| *SF3B1* | | 1.081 | 0.702-1.664 | 0.724 | |  |  |  |  | |  |  |  |  |
| *SRSF2* | | 1.705 | 0.953-3.052 | 0.072 | |  | 2.193 | 1.203-4.000 | 0.01 | |  |  |  |  |
| *TET2* | | 1.844 | 1.217-2.795 | 0.004 | |  | 2.259 | 1.478-3.453 | <0.001 | |  |  |  |  |
| *SETBP1* | | 1.653 | 1.033-2.647 | 0.036 | |  | 1.647 | 1.014-2.673 | 0.044 | |  | 2.148 | 1.215-3.795 | 0.008 |
| *TP53* | | 2.915 | 1.967-4.320 | <0.001 | |  | 3.067 | 2.052-4.582 | <0.001 | |  |  |  |  |
| *NRAS* | | 1.837 | 1.134-2.976 | 0.014 | |  |  |  |  | |  |  |  |  |
| *PTPN11* | | 2.481 | 1.350-4.559 | 0.003 | |  | 2.077 | 1.105-3.905 | 0.023 | |  | 2.009 | 1.011-3.989 | 0.046 |

HR, hazard ratio; CI, confidence interval.
